# CXCR4-Directed Imaging in Solid Tumors

**DOI:** 10.3389/fonc.2019.00770

**Published:** 2019-08-14

**Authors:** Rudolf A. Werner, Stefan Kircher, Takahiro Higuchi, Malte Kircher, Andreas Schirbel, Hans-Jürgen Wester, Andreas K. Buck, Martin G. Pomper, Steven P. Rowe, Constantin Lapa

**Affiliations:** ^1^Department of Nuclear Medicine, University of Wuerzburg, Wuerzburg, Germany; ^2^Comprehensive Heart Failure Center, University of Wuerzburg, Wuerzburg, Germany; ^3^Department of Nuclear Medicine, Hanover Medical School, Hanover, Germany; ^4^Institute for Pathology, University of Wuerzburg, Wuerzburg, Germany; ^5^Graduate School of Medicine, Dentistry and Pharmaceutical Sciences, Okayama University, Okayama, Japan; ^6^Pharmaceutical Radiochemistry, Technische Universität München, Munich, Germany; ^7^Division of Nuclear Medicine and Molecular Imaging, The Russell H. Morgan Department of Radiology and Radiological Science, Johns Hopkins University School of Medicine, Baltimore, MD, United States; ^8^Department of Urology, The James Buchanan Brady Urological Institute, Johns Hopkins University School of Medicine, Baltimore, MD, United States

**Keywords:** CXCR4, [^68^Ga]Pentixafor, theranostics, solid tumors, chemokine receptor

## Abstract

Despite histological evidence in various solid tumor entities, available experience with CXCR4-directed diagnostics and endoradiotherapy mainly focuses on hematologic diseases. With the goal of expanding the application of CXCR4 theranostics to solid tumors, we aimed to elucidate the feasibility of CXCR4-targeted imaging in a variety of such neoplasms.

**Methods:** Nineteen patients with newly diagnosed, treatment-naïve solid tumors including pancreatic adenocarcinoma or neuroendocrine tumor, cholangiocarcinoma, hepatocellular carcinoma, renal cell carcinoma, ovarian cancer, and prostate cancer underwent [^68^Ga]Pentixafor PET/CT. CXCR4-mediated uptake was assessed both visually and semi-quantitatively by evaluation of maximum standardized uptake values (SUV_max_) of both primary tumors and metastases. With physiologic liver uptake as reference, tumor-to-background ratios (TBR) were calculated. [^68^Ga]Pentixafor findings were further compared to immunohistochemistry and [^18^F]FDG PET/CT.

**Results:** On [^68^Ga]Pentixafor PET/CT, 10/19 (52.6%) primary tumors were visually detectable with a median SUV_max_ of 5.4 (range, 1.7–16.0) and a median TBR of 2.6 (range, 0.8–7.4), respectively. The highest level of radiotracer uptake was identified in a patient with cholangiocarcinoma (SUV_max_, 16.0; TBR, 7.4). The relatively low uptake on [^68^Ga]Pentixafor was also noted in metastases, exhibiting a median SUV_max_ of 4.5 (range, 2.3–8.8; TBR, 1.7; range, 1.0–4.1). A good correlation between uptake on [^68^Ga]Pentixafor and histological derived CXCR4 expression was noted (*R* = 0.62, *P* < 0.05). In the 3 patients in whom [^18^F]FDG PET/CT was available, [^68^Ga]Pentixafor exhibited lower uptake in all lesions.

**Conclusions:** In this cohort of newly diagnosed, treatment-naïve patients with solid malignancies, CXCR4 expression as detected by [^68^Ga]Pentixafor-PET/CT and immunohistochemistry was rather moderate. Thus, CXCR4-directed imaging may not play a major role in the management of solid tumors in the majority of patients.

## Introduction

C-X-C motif chemokine receptor 4 (CXCR4) is overexpressed in more than 20 tumor types and plays a crucial role in tumor growth, tumor invasiveness, cancer cell-microenvironment interaction, and metastasis (1, 2). Notably, the presence of CXCR4 has been linked to unfavorable outcomes in multiple different tumor entities, including hematologic malignancies, breast cancer, renal cell carcinoma, gynecologic malignancies, pancreatic adenocarcinoma, and hepatocellular carcinoma (3).

[^68^Ga]Pentixafor is a radiolabelled CXCR4 ligand that allows for sensitive and high-contrast visualization of the presence of the receptor *in vivo* (4, 5). Its use for non-invasive whole-body positron emission tomography (PET) imaging has been demonstrated in multiple (mainly hematologic) malignancies and also inflammatory disease conditions (6–14). Additionally, ^90^Y- or ^177^Lu-labeled Pentixather (15), the therapeutic partner of [^68^Ga]Pentixafor, has successfully been introduced for the treatment of hematologic neoplasias such as multiple myeloma, diffuse large B cell lymphoma, and acute myeloid leukemia (16–20).

In solid malignancies, pilot studies have hinted at a role for CXCR4-directed imaging in various selected diseases, such as small cell lung cancer, esophageal adenocarcinoma, and poorly differentiated neuroendocrine neoplasms (21–23). Of note, Blümel et al. reported on the use of [^68^Ga]Pentixafor in patients diagnosed with adrenocortical cancer, with 70% of the subjects being potentially suitable for a treatment with [^177^Lu]/[^90^Y]Pentixather (24).

On the other hand, Vag et al. could not detect relevant [^68^Ga]Pentixafor uptake in a heterogenous subset of different solid cancers, including non-small cell lung cancer, malignant melanoma, sarcoma, cancer of unknown primary (CUP), or breast cancer (25). Our group reported on rather discouraging results in a small cohort of patients with malignant pleural mesothelioma (26).

Thus, given these contradictory findings among different solid tumor entities, we aimed to broaden the experience of CXCR4-targeted PET imaging in solid cancers by investigating a subset of different tumors, including cholangiocarcinoma (CCC), hepatocellular carcinoma (HCC), pancreatic adenocarcinoma or neuroendocrine tumor, and ovarian cancer.

## Materials and Methods

[^68^Ga]Pentixafor was administered on a compassionate use basis in compliance with §37 of the Declaration of Helsinki and The German Medicinal Products Act, AMG §13.2b. All patients underwent imaging for clinical purposes and gave written and informed consent to the diagnostic procedures. The local institutional review board waived the requirement for additional approval because of the retrospective character of this study.

### Patients

Between September, 2014 and August, 2015, 19 patients (11 males, 8 females; aged 71 ± 7 years; range, 60–81 y) with newly diagnosed, treatment-naive solid tumors underwent [^68^Ga]Pentixafor-PET/computed tomography (CT) for assessment of CXCR4 expression. In 3/19 (15.8%) subjects, additional [^18^F]FDG PET/CT was also performed for staging purposes within 2 weeks after [^68^Ga]Pentixafor-PET (interval between both scans, median 8 days; range, 1–12 d).

Following imaging, 17/19 (89.5%) subjects underwent either tumor biopsy (8/17, 47.1%) or surgery (*n* = 9/17, 52.9%) after a median of 4.5 days (range, 1–55 d). Table 1 gives an overview of the clinical information for this patient cohort.

**Table 1:** Overview of included subjects.

**Patient no**.	**Tumor entity**	Grading*	TNM*	**Visually detectable lesions on PET**	**Primary on [**^****68****^**Ga]Pentixafor PET**			**Metastases on [**^****68****^**Ga]Pentixafor PET**			**IRS**
					**SUV_**max**_**	**TBR**	**VD**	**SUV_**max**_**	**TBR**	**VD**	
1	CCC	G2	–	1	4.65	1.68	0	–	–	0/1	0
2	RCC	G2	pT1b pNX Mx	1	1.88	0.88	0	–	–	0/1	1
3	Pancreas (NET)	G1	–	3	5.77	2.68	1	2.92	1.36	2/3	9
4	Pancreas (NET)	G2	–	>5	4.83	1.74	1	–	–	0/5	3
5	PDAC	–	pT3 Nx Mx	1	7.13	2.57	0	–	–	0/1	4
6	CUP	–	–	3	–	–	0	2.33	0.96	3/3	0
7	CCC	–	Tx Nx M1	3	16	7.41	1	8.83	4.09	2/3	12
8	HCC	–	–	0	4.39	2.71	0	–	–	–	–
9	Ovarian (low-grade serous)	–	pT2b N1 Mx	2	9.41	4.22	1	3.84	1.72	1/2	6
10	Prostate	–	–	0	1.7	0.85	0	–	–	–	–
11	PDAC	–	–	0	7.58	3.72	1	–	–	–	9
12	CCC	G3	pT1 pNx Mx	2	12.09	5.02	1	6.67	2.77	1/2	2
13	HCC	G3	pT2 pNx Mx	1	4.97	3.88	1	–	–	0/1	0
14	PDAC	–	–	1	8.22	2.07	1	–	–	0/1	6
15	Ovarian (granulosa cell tumor)	–	pT1a pNx Mx	1	2.69	1.48	0	–	–	0/1	0
16	PDAC	G2	pT2 pN1 Mx	>5	9.22	3.35	1	4.45	1.62	1/5	2
17	HCC	G3	pT1 pN0 Mx	0	3.74	1.33	0	–	–	0/1	0
18	Ovarian (high-grade serous)	G3	pT3c Nx Mx	>5	2.26	1.06	0	–	–	0/5	3
19	CCC	–	–	4	6.48	3.54	1	7.84	4.11	4/4	2

PET, positron emission tomography; IRS, immunoreactive score; SUV_max_, maximum standardized uptake value; TBR, tumor to background ratio; VD, visual detectability; CCC, cholangiocellular carcinoma; RCC, renal cell carcinoma; NET, neuroendocrine tumor; PDAC, pancreatic ductal adenocarcinoma; CUP, cancer of unknown primary; HCC, hepatocellular carcinoma. All patients underwent [^68^Ga]Pentixafor PET/CT, while in 3/19 subjects, additional [^18^F]FDG PET/CT was performed. *prior to CXCR4-directed imaging.

### Synthesis of [^68^Ga]Pentixafor

^68^Ga]Pentixafor was synthesized as previously described using a fully GMP compliant automated synthesizer (GRP, Scintomics, Fürstenfeldbruck, Germany) (27).

### PET Imaging

All PET/CT scans were performed on a dedicated PET/CT scanner (Siemens Biograph mCT 64; Siemens Healthineers, Erlangen, Germany). Before acquisition of [^18^F]FDG PET, patients fasted for at least 6 h and blood glucose levels were <160 mg/dl. Prior to [^68^Ga]Pentixafor, patients did not fast. Imaging was performed 60 min after administration of 64–166 MBq (median, 145 MBq) of [^68^Ga]Pentixafor and 297 (*n* = 1) or 301 (*n* = 2) MBq of [^18^F]FDG, respectively. Spiral CT with (dose modulation with a quality reference of 210 mAs) or without (80mAs) intravenous contrast (120 kV, 512 × 512 matrix, 5 mm slice thickness) including a field of view from the base of the skull to the proximal thighs was acquired. Consecutively, PET emission data were acquired in three-dimensional mode with a 200 × 200 matrix with 2–3 min emission time per bed position. After decay and scatter correction, PET data were reconstructed iteratively with attenuation correction using the algorithm implemented by the manufacturer (Siemens Esoft, Siemens Healthineers, Erlangen, Germany).

### Image Analysis

All PET/CT studies were visually assessed by two experienced nuclear medicine physicians (RAW and CL). Lesions were rated as visually detectable if they could be identified by both reviewers on the PET images in a consensus setting.

For derivation of maximum standardized uptake values (SUV_max_) of both primary tumors (all patients) and metastatic disease (if present, in *n* = 7/19, 36.8%), 3-dimensional volumes of interest (VOI) were drawn around the respective lesions. If patients displayed more than 5 metastases (all histologically proven or verified by imaging follow-up), the five lesions with the highest uptake were selected. For calculation of tumor-to-background ratios (TBR), VOIs with a diameter of 3 cm were placed in normal liver parenchyma and mean SUV were noted. The radiotracer concentration in the VOI was normalized to the injected dose per kilogram of patient's body weight to derive the SUVs (22).

For [^18^F]FDG-PET/CT, an analogous procedure was carried out.

### Histological Tumor Characterization

Immunohistochemistry was performed on 10% formalin fixed paraffin embedded tissue sections (3 μm) and scored as previously described (28). CXCR4-immunohistochemistry was conducted using an anti-CXCR4 rabbit polyclonal antibody (ab2074; Abcam, Cambridge, United Kingdom) followed by detection with the DAKO *en vision* system according to the manufacturer's protocol. All immunostained sections were counterstained for 3 min with hematoxylin. The analysis of the stained sections was done semi-quantitatively by light-microscopy according to the immunoreactive score (IRS) by Remmele and Stegner (29). The percentage of CXCR4-positive cells was scored as follows: 0 (no positive cells), 1 (<10% positive cells), 2 (10–50% positive cells), 3 (>50–80% positive cells), and 4 (>80% positive cells). Additionally, the intensity of staining was graded: 0 (no color reaction), 1 (mild reaction), 2 (moderate reaction), 3 (intense reaction). Multiplication of both scores for a given sample yields the IRS classification: 0–1 (negative), 2–3 (mild), 4–8 (moderate), 9–12 (strongly positive). For a more detailed description please refer to Werner et al. (22). IRS were correlated with imaging findings.

### Statistical Analysis

Descriptive statistics were predominantly utilized. All results are displayed as mean ± SD or as median + range where appropriate. The two-tailed paired Student's *t*-test was used to check for a correlation between [^68^Ga]Pentixafor SUV_max_ and histologic CXCR4 expression. A *P*-value of <0.05 was considered to be statistically significant.

## Results

### Clinical Findings

All patients presented with newly diagnosed, treatment-naïve solid tumors. The following histologic entities were represented in the cohort: pancreatic ductal adenocarcinoma (*n* = 4), pancreatic neuroendocrine tumor (*n* = 2, initially suspected as adenocarcinoma), CCC (*n* = 4), HCC (*n* = 3), and ovarian cancer (*n* = 3, one low- and one high-grade serous carcinoma as well as one granulosa cell tumor of the ovary, respectively). The remaining three subjects had, renal cell carcinoma (RCC), prostate cancer, and CUP, respectively. Evidence of metastases was detected in 7/19 (36.8%) patients.

Patient details are provided in Table 1.

### Imaging Results of [^68^Ga]Pentixafor PET/CT

On a visual basis, 10/19 (52.6%) primary tumors and 14/49 (28.6%) metastases were detectable. In semi-quantitative analysis, the median SUV_max_ of the primary was 5.4 (range, 1.7–16.0) with a median TBR of 2.6 (range, 0.8–7.4). The highest SUV_max_ were identified in patients suffering from CCC (#7, SUV_max_, 16.0; #12, SUV_max_, 12.1; Table 1).

Metastases exhibited a median SUV_max_ of 4.5 (range, 2.3–8.8) with a TBR of 1.7 (range, 1.0–4.1).

### Comparison of [^68^Ga]Pentixafor PET/CT With [^18^F]FDG PET/CT

In 3/19 (15.8%) patients (#3, #8, and #10), an additional [^18^F]FDG PET/CT scan was conducted. The primary tumor was identified in all subjects, while it was not visualized in 2/3 by [^68^Ga]Pentixafor (#8 and #10). In semi-quantitative assessment, both SUV_max_ and TBR were higher than on CXCR4-directed imaging: Median SUV_max_ of the primary was 14.3 (14.3, 18.0, 9.0; [^68^Ga]Pentixafor, 4.4; 5.8, 4.4, 1.7, respectively) with a median TBR of 4.8 (range, 2.9–6.7; [^68^Ga]Pentixafor, 2.7; 2.7, 2.7, 0.8, respectively).

[^18^F]FDG PET/CT detected metastases in 2/3 (#3 and #8) patients with a median SUV_max_ of 13.3 (range, 11.5–16.6) and a median TBR of 4.7 (range, 3.9–5.6) whereas [^68^Ga]Pentixafor visualized only 2 metastases in patient #3 (SUV_max_, 3.9 and 2.0, respectively).

### Immunohistochemical Assessment

A total of 17 samples could be investigated in a pathological assessment. 5/17 samples (29.4%) were rated as negative, 6/17 (35.4%) as “weakly” positive (IRS score 1–3), 3/17 (17.6%) as “moderately” positive (IRS scores 4–8), and 3/17 (17.6%) as “strongly” positive (IRS scores 9–12) (Table 1). Notably, both membranous as well as intra-cytoplasmatic staining for CXCR4 was confirmed.

**Figure 1:**
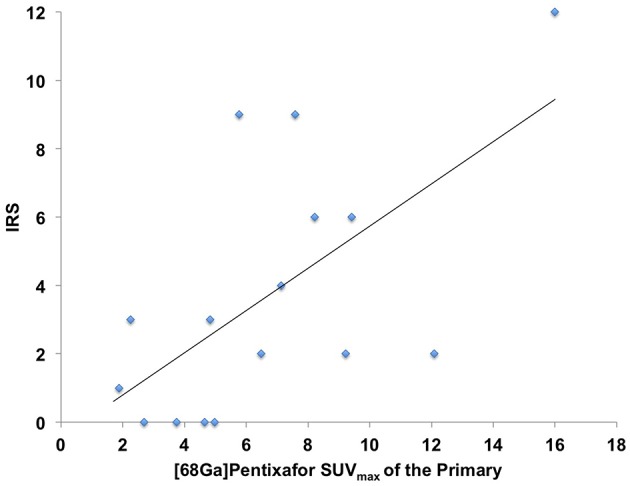
Correlation between immunoreactive score (IRS) vs. [^68^Ga]Pentixafor SUV_max_ of the primary among all tumor entities available for analysis *R* = 0.62.

**Figure 2:**
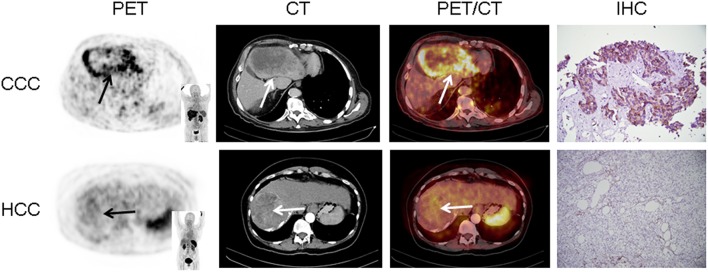
Concordance of immunohistochemistry (IHC) and non-invasive CXCR4-directed positron emission tomography (PET) imaging in patients suffering from cholangiocellular carcinoma (CCC, upper row, patient #7) and hepatocellular carcinoma (HCC, lower row, patient #17). Upper row (patient #7): Display of transaxial PET (left), computed tomography (CT, second panel) and fused PET/CT (third panel) images of a left liver lesion. The lesion demonstrates high CXCR4 expression, which could be confirmed in the surgical specimen after tumor resection (IHC, fourth panel). The immunoreactive score (IRS) was 12 (Table 1). Lower row (patient #17): Display of transaxial PET (left), CT (second panel) and fused PET/CT (third panel) images of the primary. The lesions demonstrates no CXCR4 expression on PET. The patient presented with negative IHC for CXCR4 derived from a surgical specimen (IRS, 0, Table 1). Magnification of IHC: × 400.

**Figure 3:**
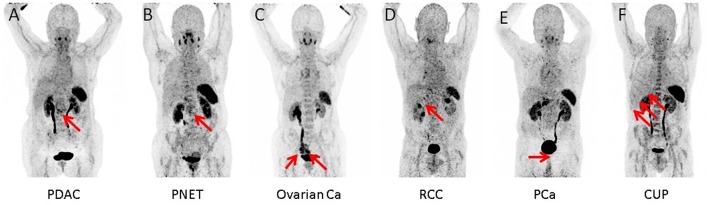
Maximum intensity projections of CXCR4-directed positron emission tomography of other selected tumor entities, primarily demonstrating moderate to no uptake on CXCR4-directed imaging (arrows). Pancreatic ductal adenocarcinoma [PDCA; **(A)**], pancreatic neuroendocrine tumor [PNET; **(B)**], ovarian cancer **(C)**, renal cell carcinoma [RCC; **(D)**], prostate cancer [PCA; **(E)**], and cancer of unknown primary (CUP) with multiple liver metastases **(F)**. Only the tumor masses of the patient with low-grade ovarian carcinoma (patient #9) display relevant CXCR4 expression.

In 16/17 (94.1%), imaging results (SUV_max_ of the primary) could be compared to immunohistological CXCR4 staining, while in 1/17 (5.9%) a SUV_max_ could not be derived as the primary could not be identified (#6, suffering from CUP). A significant correlation between IRS and [^68^Ga]Pentixafor SUV_max_ of the primary among all tumor entities was detected (R = 0.62, *P* < 0.05, Figure 1). Figure 2 shows concordant cases of immunohistochemistry and non-invasive imaging in patients suffering from CCC (patient #7) and HCC (patient #17), respectively. Figure 3 demonstrates CXCR4-directed imaging in further selected tumor entities which (with the exception of patient #9 suffering from low-grade ovarian carcinoma) primarily demonstrated moderate to no uptake on CXCR4-directed imaging.

## Discussion

An extensive body of literature has demonstrated that over-expression of CXCR4 is linked with increased aggressiveness and worse prognosis in solid cancers. Thus, this chemokine receptor is an interesting target in oncology and several therapeutic antagonists have been developed (30, 31). A recent phase I trial in women with advanced HER-2 negative metastatic breast cancer investigated a combination regimen of balixafortide (a peptidic CXCR4 antagonist) and eribulin and demonstrated favorable safety and tolerability as well as promising anti-tumor activity (32). Additionally, a first experience with chemokine receptor-directed radioligand therapy in heavily pre-treated hematologic disease has been reported (17–19).

As an important pre-requisite to CXCR4 directed therapy, [^68^Ga]Pentixafor PET/CT enables the non-invasive evaluation of receptor expression of all tumor lesions. Marked inter- and intra-individual differences in CXCR4 expression have been noted (33). Further, the receptor presentation on the tumor cell surface seems to be highly dynamic and influenced by a variety of factors including previous therapy (34).

In this study, we aimed to expand the experience with non-invasive imaging of CXCR4 in solid cancers. Previous reports had hinted at a potential role of chemokine-directed imaging in selected entities such as small cell lung cancer, adrenocortical carcinoma, or glioblastoma (21, 24, 35), while another study by Vag et al. questioned its suitability in other tumors including sarcoma, pancreatic cancer, and breast cancer (25). In the present analysis, additional solid cancers in which CXCR4 expression had been linked to metastasis and inferior outcomes such as renal cell cancer (36), ovarian cancer (37), and CCC (38) were investigated. All patients presented with newly diagnosed, treatment-naïve disease. To exclude potential influence of concomitant therapy on receptor surface expression, biopsies, and/or surgery were performed shortly after PET imaging and prior to treatment initiation. However, only weak to moderate [^68^Ga]Pentixafor uptake was recorded in the vast majority of patients and almost 80% of lesions could not be visually detected. In addition, only two patients exhibited primary tumor SUV_max_ > 10. Interestingly, both patients suffered from CCC (#7, Figure 2 and #12). These findings were paralleled by immunohistochemistry that also identified relevant CXCR4 expression in few tumor specimens and correlated well with non-invasive imaging results (*R* = 0.62, Figure 1).

Taken together, the current findings indicate that [^68^Ga]Pentixafor is unlikely to play a major role in staging and re-staging of most solid tumors, in particular when compared to [^18^F]FDG. Given the physiologic expression of CXCR4 on hematopoietic stem cells and thus the need for subsequent stem cell support (17, 18), endoradiotherapy with radiolabelled Pentixather might also be reserved to very selected cases.

Future efforts for potential applications of CXCR4-directed imaging might focus on the characterization of intra-/inter-lesional heterogeneity by performing dual-radiotracer studies (in conjunction with [^18^F]FDG) to visualize different levels of tumor de-differentiation and predict lesions with prognostic relevance. For example, CXCR4-directed PET/CT might help to visualize receptor positive cancer stem cells (and their niche) which are considered to be especially resistant to radiation or chemotherapy (1, 39).

Several limitations of the present study have to be considered. It is retrospective and the number of patients is rather small. Further research including a higher number of subjects is definitely warranted to confirm the preliminary findings of the present feasibility study. Histologic validation of imaging results was not available in all cases. Additionally, a variety of different tumor entities were included. However, all patients presented with newly diagnosed disease and were treatment-naïve at the time of imaging. Moreover, in only 3 patients, an additional [^18^F]FDG PET/CT has been conducted and future studies should evaluate potential tumor heterogeneity in a higher number of subjects. Although the value of [^68^Ga]Pentixafor PET/CT has been investigated before, our small series adds first experience with other tumor entities such as ovarian cancer and renal cell carcinoma, along with histopathologic proof in the majority of the cases.

## Conclusions

In this cohort of various treatment-naïve solid malignancies, CXCR4 expression as detected by [^68^Ga]Pentixafor-PET/CT and immunohistochemistry was rather moderate. Thus, CXCR4-directed imaging may not play a major role in the management of the majority of solid cancer patients.

## Data Availability

The raw data supporting the conclusions of this manuscript will be made available by the authors, without undue reservation, to any qualified researcher.

## Ethics Statement

The local institutional review board of University Würzburg waived the requirement for additional approval because of the retrospective character of this study. All subjects gave written informed consent in accordance with the Declaration of Helsinki.

## Author Contributions

RW, SK, CL, TH, MP, and SR designed the study, wrote the manuscript, and researched data. MK, AS, H-JW, and AB performed analysis. All authors aided in drafting the manuscript and revised it critically for important intellectual content. All authors read and approved the final manuscript.

### Conflict of Interest Statement

The authors declare that the research was conducted in the absence of any commercial or financial relationships that could be construed as a potential conflict of interest.
